# Austin District Medical Society—Proceedings and Resolutions

**Published:** 1891-03

**Authors:** T. J. Bennett

**Affiliations:** Sec’y.


### Society Notes.

**The Austin District Medical Society** met in Austin on the 19th inst. and had a most interesting session. The follow-lowing delegates were elected to the Waco meeting of the State Association, April 28th, prox.:

Drs. A. Garwood, J. A. Davis, Joe Cummings, S. Cunningham, W. T. Richmond, F. R. Martin, G. W. Christian, J. S. Poyner, J. W. Hamilton, L. H. Hardy, R. P. Talley, H. H. Thrope, R. Atkinson, W. H. Holloway, B. E. Hadra, W. H. Barnwell, J. W. Carhart.

Dr. Dock, of Galveston, Pathologist of the Texas Medical College and Hospital, delivered a highly interesting and instructive lecture on *Amœbi Coli* in relation to hepatic abscess and dysentery.

Dr. Bennett, the indefatiguable Secretary of this flourishing Society deserves mnch credit for his efforts, and great success in building up so strong and influential an organization. The roll of active members now numbers 86 and embraces the leading physicians of nine adjacent counties, and even members from as far as Galveston and Temple. [Ed.]

The following resolutions were adopted; the latter by a rising vote, and unanimously:

Whereas, it is contemplated that in time the State will take control and assume the management of the Texas Medical College, converting it into the Medical Department of the University of Texas; and

Whereas, Dr. B. E. Hadra, the present incumbent of the Chair of Surgery in said Texas Medical College, is well known to us and known to be eminently qualified for the position, and we believe that he will fill the chair with credit to himself and honor to the State;

*Resolved*, That the Austin District Medical Society, in convention assembled at Austin, the 19th day of March, 1891, do hereby endorse the said Dr. Hadra as eminently qualified to teach surgery in all its branches, and recommend him to the favorable consideration of the Hon. Board of Regents for appointment to the Chair of Surgery in the Medical Department of the State University.

WHEREAS, one of our members, Dr. F. E. Daniel, has recently been made the subject of a most unjust and slanderous publication in the *Texas Health Journal*, whereby his moral and professional character has been furiously assailed; and

WHEREAS, Dr. Daniel has resided in our midst a number of years, and his daily walk has been under our observation, we know him well, in every relation of life; and

WHEREAS, we have evidence of the most satisfactory character of the purity and nobility of his character prior to his coming among us; therefore be it

*Resolved*, By the Austin District Medical Society that we repudiate said publication in every detail as *false;* and further

*Resolved*, That we, having known Dr. Daniel intimately, and been familiar with him as a citizen, a physician and a gentleman, do hereby endorse him an honorable man, a physician of ability and purity of professional life—a faithful laborer in the cause of legitimate medicine, a worthy member—and one of the organizers of this and other Societies; and as a Christian gentleman, whose walk in life is, and has been, above reproach.

*Resolved*, That the attention of the Texas State Medical Association is hereby called to the said publication.

*Resolved*, That these resolutions be published with the proceedings of this Society, and that they be spread upon its minutes.

Adapted unanimously by rising vote.

March 19, 1891. T. J. BENNETT, M. D., Sec'y.

[A true copy.]

